# Epigenetics of functional hypothalamic amenorrhea

**DOI:** 10.3389/fendo.2022.953431

**Published:** 2022-08-11

**Authors:** L. Fontana, E. Garzia, G. Marfia, V. Galiano, M. Miozzo

**Affiliations:** ^1^ Medical Genetics, Department of Health Sciences, Università degli Studi di Milano, Milan, Italy; ^2^ Unit of Medical Genetics, ASST Santi Paolo e Carlo, Milan, Italy; ^3^ Reproductive Medicine Unit, Department of Mother and Child, San Paolo Hospital, ASST Santi Paolo e Carlo, Milan, Italy; ^4^ Aerospace Medicine Institute “A. Mosso”, Italian Air Force, Milan, Italy; ^5^ Laboratory of Experimental Neurosurgery and Cell Therapy, Neurosurgery Unit, Fondazione IRCCS Ca’ Granda Ospedale Maggiore Policlinico, Milan, Italy

**Keywords:** functional hypothalamic amenorrhea (FHA), epigenetics, susceptibility genes, anorexia nervosa, delayed puberty

## Abstract

Functional hypothalamic amenorrhea (FHA) is a temporary infertility characterized by the suppression of the hypothalamic–pituitary–gonadal (HPG) axis, induced by the inhibition of the hypothalamic pulsatile secretion of the gonadotropin-releasing hormone (GnRH), in the presence of stressors, including eating disorders, excessive exercise, and psychological distress. Although the stressful factors that may lead to FHA are well-established, little is known about the inter-individual variability in response to stress and the consequent inhibition of the HPG axis. Not all women, indeed, manifest FHA in presence of stressful conditions. Recent studies highlighted a genetic contribution to FHA. Rare or polymorphic variants in genes that control the development and/or function of GnRH neurons may contribute, indeed, to the adaptability of the reproductive axis to stress factors. Also epigenetic changes have been associated with different pathways involved in the HPG axis and therefore, take part in FHA and confer a personal predisposition to anovulation consequent to a stressful event, or represent biological markers of response to stress. This review summarizes recent advances in the identification of the contribution of (epi)genetics to FHA and to long-term complications of functional amenorrhea, and reports insights into the involvement of additional genetic loci in FHA development on the bases of the clinical and molecular overlap with other gynecological and/or psychological conditions. Finally, we describe the promising application of induced pluripotent stem cells (iPSCs) as a new approach to investigate the molecular pathways involved in FHA.

## Introduction

Functional hypothalamic amenorrhea (FHA) may be considered a natural protective mechanism in women against stressful events, that temporarily suppress reproductive functions when physical conditions are not suitable to sustain a pregnancy. FHA is characterized by the suppression of the hypothalamic–pituitary–gonadal (HPG) axis, induced by the inhibition of the hypothalamic pulsatile secretion of the gonadotropin-releasing hormone (GnRH), in the presence of stressors, including eating disorders, excessive exercise, and psychological distress. FHA thus delineates as a temporary infertility characterized by the absence of menses for at least 3-6 consecutive months in absence of pregnancy, hyperandrogenism, hyperprolactinemia or other endocrine dysfunctions.

Although the stressful factors that may lead to FHA are well-established, little is known about the inter-individual variability in response to stress and the consequent inhibition of the HPG axis. Studies in women identified a high variability in the occurrence of FHA after heavy training. In particular, the frequency of FHA in athletes ranges from 6 to 43% (1); while in non-athletes, following the same training scheme of athletes, the frequency of FHA has been reported to be higher, with only about 14% of women with regular menstrual cycles during the training (2). Studies in nonhuman primates also confirmed the variable response of the HPG to stress (3–5). Like women, female macaques exposed to a combination of stresses showed different degrees of anovulation. Some animals, considered stress-sensitive, have an immediate suppression of ovulation and menstrual cycles, whereas others are highly stress-resilient and never experience FHA in presence of stress (3). Immunohistochemistry analysis of hypothalamic sections from resilient and stress-sensitive monkeys highlighted a higher GnRH expression in the neuron soma and lower GnRH levels in the neuron fibers of stress-sensitive animals, suggesting differences in the neuronal mechanisms involved in GnRH synthesis, transport and release in stress-sensitive compared with resilient animals (5).

Taken together, these studies suggest that the genetic variability in genes that control the development and/or function of GnRH neurons may contribute to the adaptability of the reproductive axis to a stressful condition. This hypothesis is confirmed by recent studies that highlighted the presence of a higher frequency of rare variants in genes associated with idiopathic hypogonadotropic hypogonadism (IHH) in women with FHA compared to women with regular menses (6, 7). The identification of other pathways, comprising those involving ghrelin and leptin that control the HPG axis as endocrine mediators of energy balance, could suggest the classification of FHA as a multifactorial and complex condition.

In addition to the presence of rare variants and polymorphisms that may alter the regulation of the axis, epigenetic changes could be associated with different pathways involved in the HPG axis (8, 9) and therefore, take part in FHA and confer a personal predisposition to anovulation consequent to a stressful event, or represent biological markers of response to stress.

In this review we summarize recent advances in the identification of the contribution of (epi)genetics to FHA and to long-term complications of functional amenorrhea, and reports insights into the involvement of additional genetic loci in FHA development on the bases of the clinical and molecular overlap with other gynecological and/or psychological conditions. Finally, we describe the promising application of induced pluripotent stem cells (iPSCs) as a new approach to investigate the molecular pathways involved in FHA.

## Genetics and molecular overlapping between FHA and IHH

The clinical overlapping between FHA and IHH prompted the hypothesis that mutations in genes involved in IHH may confer susceptibility to the functional deficiency of GnRH secretion, the hallmark of FHA. IHH is, indeed, characterized by the failure to activate the pulsatile secretion of GnRH or the defective action of GnRH at pituitary level, thus resulting in absence of puberty and infertility. IHH is classified in IHH associated to anosmia, also known as Kallman syndrome (KS), a mendelian condition characterized by locus heterogeneity, that is found in about the 60% of IHH patients (10, 11); and normosmic IHH (nIHH) observed in the remaining patients.

The presence of total or partial loss of olfaction is caused by the common embryonic origin and developmental pathways of GnRH and olfactory neurons. Similar to olfactory fibers, GnRH neurons originate in the nasal placode from where they migrate to the hypothalamus (12). This common developmental pathway accounts for the defective migration of both GnRH and olfactory neurons in KS. The identification of the underlying molecular mechanism causative of KS, and the genetic characterization of affected families, allowed the identification of mutations in genes involved in the migration of GnRH and olfactory neurons, such as *KAL1* and *FGFR1* (13). More recently, mutations in the *PRORK2* and *PROK2* genes, that encode for a G-coupled receptor and its ligand with a fundamental role in the development of GnRH neuronal progenitors, have also been associated to IHH (14). Other genes that are involved in the development of IHH include: i) *CHD7*, encoding for a chromodomain protein associated to the CHARGE syndrome, suggesting a link between IHH, chromatin remodeling and transcription regulation; ii) *KISS1* and *KISS1R*, that encode for kisspeptin 1 and its endogenous receptor, that are the most potent regulators of GnRH secretion in humans; iii) *LEP*, that encodes for leptin, a fat-released hormone that regulates food intake, energy expenditure and fertility at the hypothalamic level, and its receptor LEPR, thus linking body weight with the reproductive capability; iv) *TAC3* and *TAC3R*, that encode for neurokinin B and its receptor, suggested to have a role in GnRH secretion; v) *PCSK1*, encoding for the neuroendocrine convertase 1 (NEC1), which converts inactive peptides to bioactive molecules, including the adrenocorticotropin (ACTH); and vi) *GnRHR*, that is the main regulator of GnRH secretion.

### Rare variants in IHH-related genes in FHA

**Figure 1:**
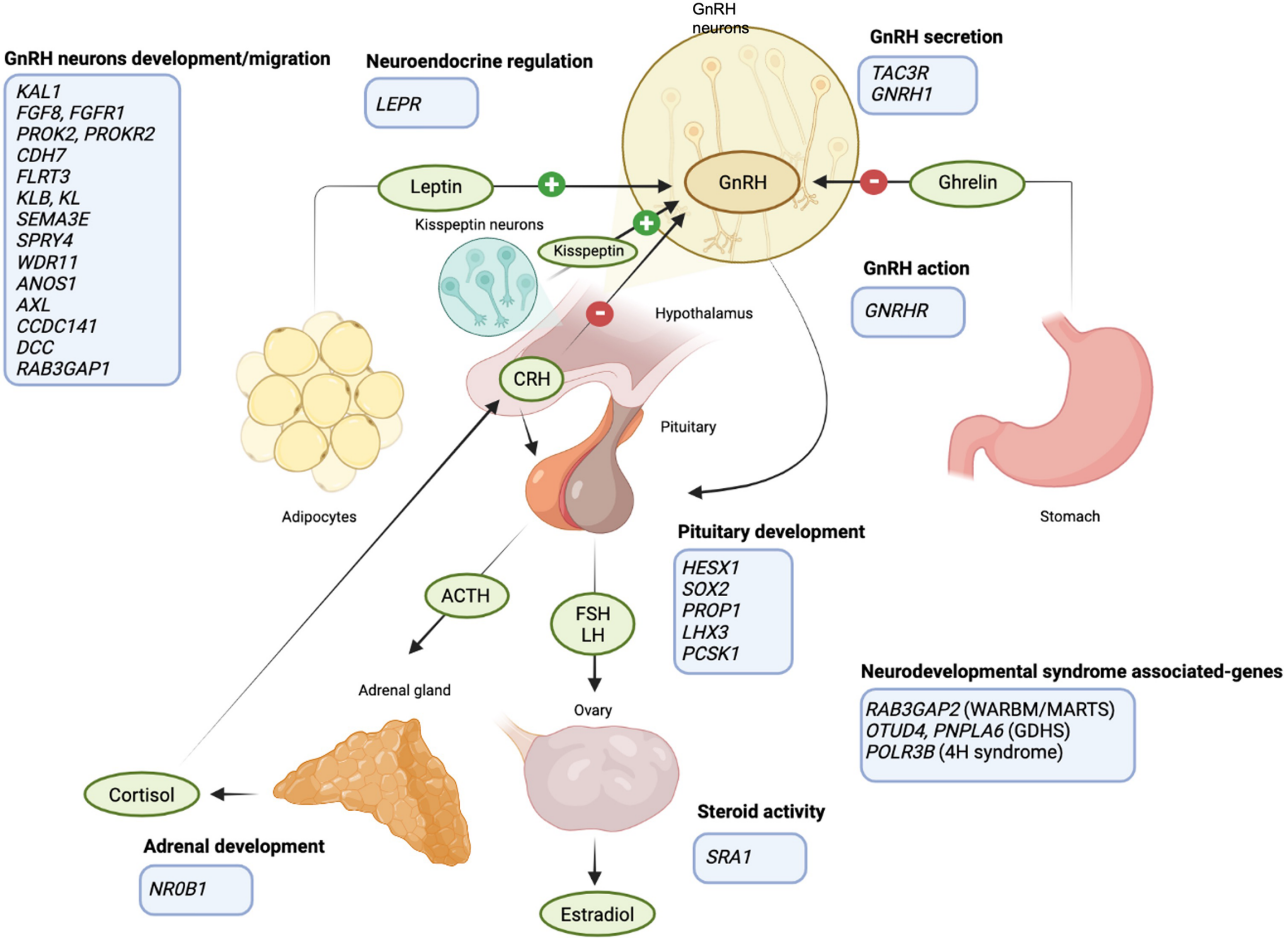
Schematic representation of the HPG axis regulation and the FHA predisposing genes. GnRH neurons in the hypothalamus release GnRH upon different stimuli: kisspeptin, produced by a specific group of hypothalamic neurons, is a major player in the neuroendocrine control of GnRH and gonadotrophins secretion; ghrelin and leptin allow the regulation of GnRH secretion according to energy balance (link between HPG axis and food intake); cortisol inhibits GnRH secretion (link between HPG axis and anxiety). FHA-predisposing genes are listed in the light blue boxes (created with BioRender.com).

After the identification of FHA in some families with KS/nIHH, Caronia and colleagues found six pathogenic variants in genes associated with IHH development in patients with FHA (**Table 1** and **Figure 1**), thus suggesting, for the first time, an involvement of rare variants in these loci in the predisposition to FHA in presence of stressful situations (6). The study included 55 women with FHA and highlighted six heterozygous variants in *FGFR1*, *PROKR2*, *GNRHR* and *KAL1* in 7 patients with FHA and at least one predisposing factor to secondary amenorrhea. Four of these patients also reported a family history of FHA, but the genetic analysis was not extended to other affected relatives to confirm the association. The observed variants were considered pathogenic, since they all were located in conserved aminoacidic residues and induced a significant loss of function (6). None of these mutations were observed in a group of 422 control women, some of them also exposed to one risk factor for FHA (i.e., training for more than 5 hours a week). In addition, one mutation in the *PROKR2* gene (L173R) and in *GNRHR* were also detected in a cohort of 160 IHH patients.

**Table 1:** Rare variants in IHH-related genes identified in FHA patients according to Caronia et al., 2011 and Delaney et al., 2020.

Gene	Protein activity	Role in HPG axis	Variants
*FGFR1*	Tyrosine kinase receptor	GnRH neuron migration/development	Arg756His
			Gly260Glu
*PROKR2*	GPCR	GnRH neuron migration/development	Arg85His
			Leu173Arg
			Thr340Ser
			Met111Arg
*GNRHR*	GPCR	GnRH action	Arg262Gln
			Ser168Agr
			Gln106Arg
*KAL1*	Cell adhesion	GnRH neuron migration/development	Val371Ile
*RAB3GAP2*	Neurotransmitter exocytosis	Neurodevelopmental syndrome with IHH*	Asp1206Tyr
			Pro527Leu
			Arg420Cys
			Leu1331Ile
*RAB3GAP1*	Neurotransmitter exocytosis	GnRH neuron migration/development	Arg336Cys
			Arg954His
*HESX1*	Transcriptional repressor	Pituitary gland development	Val129Ile
*SOX2*	Transcription factor	Pituitary gland development	Gly22Ser
*KLB*	Membrane receptor	GnRH neuron migration/development	Ala169Thr
			Gly908Val
			Lys815Glu
			Val1042Ile
*TACR3*	TAC3 receptor	GnRH secretion	His248Arg
*OTUD4*	De-ubiquitylating enzyme	Neurodevelopmental syndrome with IHH^	Pro933Arg
*SRA1*	Regulator of nuclear receptors	Steroid activity	Leu110Aspfs*25
*SPRY4*	Inhibitor of MPAK receptor	GnRH neuron migration/development	Ser241Tyr
			Gly92Val
			Cys209Tyr
*PROP1*	Transcription factor	Pituitary gland development	Ala142Val
*SEMA3E*	Growth factor	GnRH neuron migration/development	Pro171Ser
			Asn153Ser
			Asp580Asn
*GNRH1*	Gonadotropin-releasing hormone	GnRH secretion	Ile48Arg
*FGFR1*	Growth-factor receptor	GnRH neuron migration/development	Gly291Glu
*CHD7*	Chromatin-remodeling factor	GnRH neuron migration/development	Ser244Arg
			Arg459Cys
			Asp728His
			Pro1705Gln
			Arg1942Trp
			Met2527Leu
			Met396Ile
			Ser466Leu
			Leu2984Phe
			Met340Val
*LHX3*	Transcription factor	Pituitary gland development	Gly317Ser
			Arg315Pro
*WDR11*	Transcription factor	GnRH neuron migration/development	Val6Met
*POLR3B*	Subunit of RNA polymerase III	Neurodevelopmental syndrome with IHH°	Lys721*
			Arg978Cys
*KL*	Ligand of KLB	GnRH neuron migration/development	Arg751Gly
			Val845Gly
*DMXL2*	Synaptic protein	GnRH neuron migration/development	Met563Val
			Thr476Se
			Ile2573Val
			Ile1317Val
*DCC*	Transmembrane receptor	GnRH neuron migration/development	Gly470Asp
			Asp819Asn
			Val883Ile
			Asn635Ser
*PNPLA6*	Phospholipase	Neurodevelopmental syndrome with IHH^	Gly1329Arg
*AXL*	Tyrosine kinase receptor	GnRH neuron migration/development	His292Profs*47
			Gly517Ser
			Val289Met
*FLRT3*	Cell adhesion/signaling	GnRH neuron migration/development	Gln401Leu
*ANOS1*	Extracellular glycoprotein	GnRH neuron migration/development	His672Arg
			Val587Leu
			Val371Ile
			Ser511Tyr
*NR0B1*	Nuclear receptor	Adrenal gland development	Ser412Gly
*LEPR*	Leptin receptor	Neuroendocrine regulation	Val754Met
*CCDC141*	Cytoskeletal-associated protein	GnRH neuron migration/development	Glu876Lys
*PCSK1*	Protease	Pituitary gland development	Thr640Ala

Underlined variants have been observed also in eumenorrheic women.

*, Warburg Micro syndrome/Martsolf syndrome; ^, Gordon Holmes syndrome; °, 4H syndrome.

The same authors further deepened the role of genetics in FHA, extending the mutational analysis to 53 genes involved in IHH and the number of FHA patients included in the study (7). This mutational screening allowed the identification of 78 heterozygous variants in 58 out of 108 women with FHA (**Table 1** and **Figure 1**). Among these, three variants were validations of the previously smaller cohort, 32 novel variants were identified in patients resulted negative at the previously sequencing analysis, and two were detected in a patient with a previously identified mutation in the *ANOS1* gene. Most of these variants were observed in FHA women only, while few of them were also found in control women, thus excluding a pathogenetic role of these variants per se in the development of FHA. However, the frequency of rare variants in GnRH-related genes was higher in FHA patients compared to controls, and 35% of variants observed in amenorrhoeic women has been previously associated with IHH development, compared to 19% found in the control group (7). In addition, FHA women harbor a higher number of variants (three or more) compared to the control group. In this latter many participants did not show any variant. In this study, no associations with the number of FHA risk factors nor with the family history for the condition were observed (7).

Overall, this evidence further supports the involvement of genetic variants in the variable response of the reproductive axis to stress. Moreover, since only mutations in *FGFR1* and *CHD7* are known to be associated to IHH with a dominant inheritance, the authors hypothesized that the GnRH deficiency may be considered as a semi-dominant disease, with heterozygous carriers that show a milder phenotype, characterized by FHA only in presence of triggering external factors, while patients with biallelic or di/oligogenic mutations show a severe form of GnRH deficiency resulting in IHH. This is supported by the identification of some rare variants also in eumenorrheic women. These studies thus suggest, for the first time, that FHA may be part of a disease spectrum with a genetic base.

## Genetic and molecular overlapping between FHA and other gynecological disorders

Several other disorders, including delayed puberty and anorexia nervosa, share with FHA a partially overlapping clinical presentation and the causative molecular mechanisms, suggesting a common genetic base or predisposition. This common picture stems from both the central role of the hypothalamus, which controls most of the basic functions that are interconnected, and the multifunctional role of the hormones released by the hypothalamus. In particular, genetic variants in genes involved in pathways controlling appetite or stress-response might also contribute to FHA. Anorexia nervosa (AN), for example, is a multifactorial eating disorder characterized by a chronic energy deficiency that leads to the suppression of the HPG axis because of the reduced secretion of GnRH, as observed in FHA. AN and FHA enter in differential diagnosis since AN may be considered a cause of functional amenorrhea. Differently from FHA, AN shows a strong heritability since 1) family studies have demonstrated a significant prevalence of AN in first-degree relatives of probands compared to controls, with a 11.3 times more probability of relatives to develop AN compared to controls (15, 16); 2) studies on twins highlighted that monozygotic twins have a higher concordance rate of AN development compared to dizygotic twin, with an estimated heritability of 88%; 3) population studies reported an estimated heritability of approximately 58% for AN, with the remaining variance associate with environmental factors (17); 4) mutational screenings and genome-wide association studies have highlighted several genetic loci that may account for the predisposition to AN (18). Common variants in neurotrophin signaling genes, including *BDNF*, *NTRK2* and *NTRK3*, seem to contribute to the susceptibility to eating disorders (19, 20) (**Table 2**). Other signaling pathways, involving a cross-regulation of satiety and estrogen production, including the serotoninergic and leptin pathways, have been linked to an increased risk to develop AN. Common variants in the *OPRD1*, *HTRD1*, *EBF1* and *SLC6A4* genes show a higher frequency in AN patients compared to controls and segregate in family members with AN, supporting their involvement in the etiology of AN (21–24) (**Table 2**). Common or rare variants in genes involved in these pathways have never been investigated in FHA patients. However, given the close link between FHA and AN, in addition to the evidence that food restriction is one of the main risk factors for FHA development, it can be hypothesized that variants in genes involved in satiety, appetite and weight regulation may play a role in the differential response to physical and/or psychological stressors and the consequent inhibition of the HPG axis in FHA.

**Table 2:** Predisposing genes to gynecological and psychological conditions showing overlapping features with FHA, and to long-term consequences of FHA.

Disorder	Condition	Overlapping with FHA	Affected pathway	Genes involved
Gynecological disorders	Anorexia nervosa	AN is a chronic energy deficiency that leads to the suppression of the HPG axis because of the reduced secretion of GnRH	Neurotrophin signaling pathway	*BDNF* *NTRK2* *NTRK3*
			Serotoninergic and leptin pathways	*OPRD1* *HTRD1* *EBF1* *SLC6A4*
	Delayed puberty	Delayed puberty may occur in patients with FHA and can be considered an early clinical sign of this condition	IHH development	*LEPR* *GNRH1* *TACR2* *HS6ST1* *FGFR1* *KLB*
			GnRH neuron migration	*IGSF10*
Psychological disorders	Anxiety	The neuroendocrine response to stress and stress-related neuronal plasticity involves the HPG axis	Energy balance and anxiogenic effect of CRH	*NPY*
	Mood disorders	Altered neuroplasticity related to stress	Neuroplasticity, neurogenesis, neuronal survival, and differentiation	*BDNF*
Long-term consequences	Osteopenia and osteoporosis	Prolonged hypoestrogenism in FHA leads to osteopenia and osteoporosis	Estrogen receptor	*ESR1-XbaI*
			Vitamin D receptor	*VDRBsmI site* *VDRFokI site*

Delayed puberty may occur in patients with FHA and IHH and can be considered an early clinical sign of these conditions. About two-third of FHA patients report a family history of delayed puberty with an apparent autosomal dominant pattern of inheritance, sometimes with incomplete penetrance (25, 26). Genome-wide association studies highlighted an association with genes involved in IHH, including *LEPR*, *GNRH1* and *TACR2* (27, 28) (**Table 2**). Besides, genetic screenings in IHH families identified mutations in *HS6ST1*, *FGFR1* and *KLB* in IHH patients and their relatives manifesting delayed puberty only (29–31) (**Table 2**). Pathogenic mutations in the *IGSF10* gene were also found in six unrelated families with delayed puberty in a large Finnish cohort of patients (32) (**Table 2**). *IGSF10* mutations affect the migration of GnRH neurons from the vomeronasal organ in the nose to the forebrain during embryonic development, possibly affecting the predisposition to FHA as observed for other genes in the same pathway.

All this evidence supports a common genetic basis of FHA with AN, delayed puberty and IHH, strengthening, again, the hypothesis that monoallelic mutations in a subset of genes that modulate the HPG axis may lead to AN, delayed puberty and/or FHA, while biallelic mutations or a specific combination of heterozygous variants in these genes could lead to a more sever phenotype, including IHH or KS (33).

### Genetic and molecular overlapping between FHA and psychological disorders

FHA may be considered a multifactorial disease with genetic factors and pathomechanisms overlapping not only with other gynecological conditions, but also with psychiatric traits, since an anxious behavior is considered a main sign of FHA. The hyperactivation of the hypothalamic–pituitary–adrenal (HPA) axis in presence of stressing factors is, indeed, a typical feature of FHA and this is believed to be one of the most important pathogenetic factors in FHA patients (34, 35). Increased corticotropin-releasing hormone (CRH) secretion results in an augmented secretion of adrenocorticotrophin from the pituitary, and cortisol from the adrenal glands that, in turn, leads to reduced GnRH secretion.

**Figure 2:**
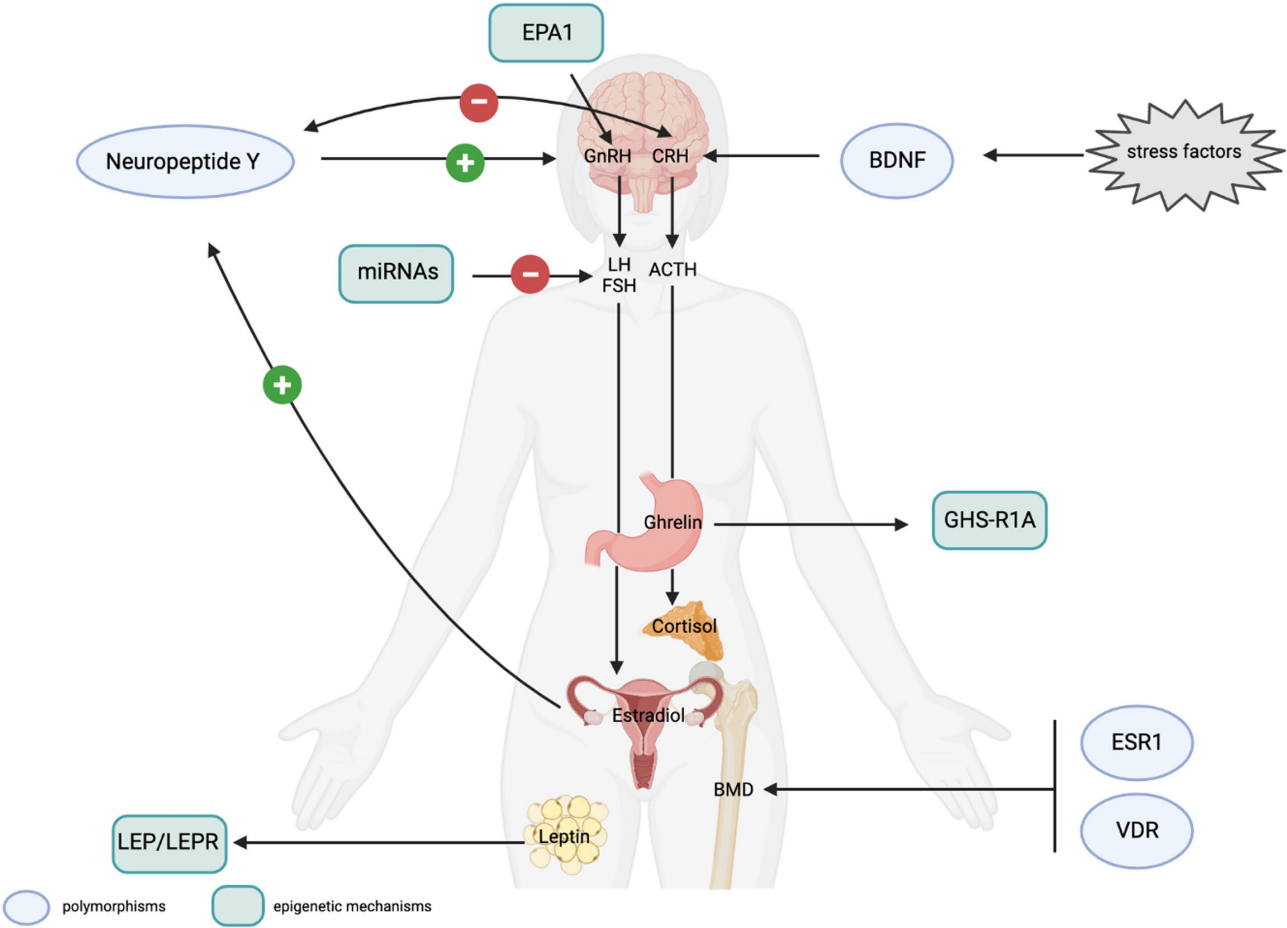
(Epi)genetic mechanisms possibly involved in FHA development and FHA-related long-term consequences. **Genetic mechanisms**. Polymorphisms in the *Neuropeptide Y* (*NPY*) and *BDNF* genes affect stress response. The NPY positively controls GnRH secretion in presence of adequate levels of estrogen and has an anxiolytic effect by counteracting CRH activity. CRH, itself, downregulates the expression of the NPY. NPY polymorphisms have been associated to resilience or stress-sensitive phenotypes. BDNF polymorphisms are suggested to affect neuroplasticity and stress responses. Polymorphisms in the estrogen receptor (ESR1) and in the vitamin D receptor (VDR) genes influence bone mineral density (BMD) and may be associated to osteopenia and osteoporosis, consequent to prolonged hypoestrogenism. **Epigenetic mechanisms**. The EPA1 transcription factor controls GnRH expression and a 5’-UTR polymorphism has been associated with a higher risk of amenorrhea in animal models. Altered methylation levels of the *LEP* and *LEPR* genes have been associated with the effect of leptin, produced by adipocytes on the HPG axis and on the personal response to psychotherapeutic treatment in AN patients. Methylation of the ghrelin receptor gene (*GHS-R1A*) are thought to be involved in ghrelin resistance affecting GnRH secretion. Specific miRNAs have been reported to control the post-transcriptional expression of LH and FSH, and to be a promising peripheral biomarkers to control the effect of hormonal therapy in FHA women. Light blue circles indicate polymorphic variants in genes possibly associated to response to stress or long-term consequences in FHA women; light green rectangles indicate the epigenetic mechanisms (including transcription factors, miRNA and methylation) that can play a role in the regulation of the HPG axis and in FHA development (created with BioRender.com).

Several studies have highlighted a strong genetic component in anxiety predisposition or resilience to stress. Genome-wide association studies identified several common genetic variants in genes involved in the neuroendocrine response to stress and in neuronal plasticity that affect stress perception and increase the risk of developing stress-related disorders. Some of these polymorphisms are in genes playing a role also in controlling the HPG axis, thus suggesting a possible link among stress-associated genetic variants, FHA, and anxiety. In particular, specific polymorphisms (i.e. rs16147 and rs3214187) in the *Neuropeptide Y,* which acts as a regulator of energy balance and counteracts the anxiogenic effect of CRH (36) (**Table 2**), have been associated to a resilience or a stress-sensitive phenotype in presence of a stressful condition (37, 38). The NPY also controls GnRH concentration, by inducing its release in presence of adequate levels of estradiol. In hypoestrogenic women, NPY inhibits GnRH release and amenorrheic patients show lower levels of basal serum NPY (39, 40). NPY may, thus, act as a link between the HPG and HPA axis, and common or rare variants in the *NPY* gene may affect the HPG axis response to stress (**Figure 2**).

Neuroplasticity has a primary role in the predisposition to stress-related disorders. According to the neurotrophic hypothesis, mood-disorders may be associated with impaired structural plasticity and cellular resilience, with a key role of the brain derived neurotrophic factor (BDNF), that is highly expressed in the hippocampus and involved in neuroplasticity, neurogenesis, neuronal survival, and differentiation (41). Several studies have highlighted an increase of BDNF serum levels associated with stress and mood disorders (42). Genome-wide association studies pointed for the involvement of the Val66Met (rs6265) in anxiety and response to stress, with Val/Val homozygotes that show increased anxiety (43), suggesting a role of BDNF in gene-environment interactions (**Table 2**). Despite the function of BDNF in FHA is not fully understood, a significant lower concentration of plasma BDNF was found in FHA patients in comparison to healthy controls (44), possibly suggesting the contribution of BDNF in the altered stress response associated to FHA (**Figure 2**), paving the way for the analysis of common variants in the BDNF gene that may account for the anxiogenic phenotype of FHA women.

## Genetic predisposition to long-term complications of FHA

FHA is a complex clinical condition, that should be addressed with a multidisciplinary approach.

The close interconnections between the hormonal and neuroendocrine status to regulate homeostasis, lead, consequently, to clinical manifestations other than amenorrhea in FHA women. The final endocrinological consequence of GnRH impairment is, indeed, hypoestrogenism. Persistent low levels of estrogen have negative effects on different aspects of female health. In particular, normal estrogen levels are fundamental in young women for bone metabolism, the correct function of the cardiovascular system, and for mental health. Considering these pleiotropic effects of hypoestrogenism, genetic studies on amenorrheic patients should also include the genotyping of candidate loci associated with long-term complications of FHA.

Among these, prolonged hypoestrogenism in young women with FHA is associated with osteopenia and osteoporosis. Studies in women with AN highlighted the association of specific polymorphisms with bone mineral density (BMD). In particular, the A allele at a polymorphic site of the estrogen receptor alpha ESR1-XbaI (rs934079) has been associated with a reduced BMD (45) (**Table 2**); while the AA genotype at the VDRBsmI site (rs1544410) and the CT genotype at the VDRFokI site (rs2228570) of the vitamin D receptor (VDR), both show a positive correlation with BMD in patients with AN (46) (**Table 2**). According to this evidence, the identification of FHA women at higher risk for reduced BMD should be mandatory for a timely treatment and to avoid long-term consequences of FHA. Polymorphic variants in ESR receptors may also be investigated in association to other FHA-related health consequences, including cardiovascular disorders primarily derived from endothelial dysfunction secondary to hypoestrogenism (47) (**Figure 2**).

Serum sex steroid levels in women also control mood. Low levels of estrogen in FHA women are strongly coupled to the modulation of the activity of different neuropeptides and neurotransmitters, in particular serotonin and dopamine, affecting mood in amenorrheic patients (48).

Mood disorders and anxiety are both causes and effects of FHA, thus establishing a negative loop that exacerbates the effects of mood disorders and sustains FHA. As previously described, many variants have been identified in genes associated with a personal response to stress and estrogen sensitivity that influence mood disorder predisposition. The identification of genetic variants in these or other genes in the same pathways may allow the identification of FHA patients who are more predisposed to mood disorders. This approach could improve a tailored therapeutic approach based on targeted therapies to avoid or disrupt the loop between FHA and mood disorders.

## The epigenetic contribution to FHA

Epigenetic mechanisms govern gene expression without changing gene sequence and comprise chromatin changes, DNA methylation, and the expression of non-coding RNAs. The epigenetic signatures can be modified in response to environmental factors, stress, or disease, and mediate the response of the organism to external factors. Importantly, they can be reverted by epigenetic drugs, as demonstrated in cancer, by changes in behavioral habits or non-pharmacological treatments, such as psychotherapy.

Recent evidence highlighted the importance of the epigenetic regulation of GnRH expression by a network of miRNAs, epigenetic modifications, and transcription factors, suggesting an important role of these mechanisms in regulating the HPG axis and, therefore, their possible involvement in FHA. The tight interplay between genes and environment in FHA development and the identification of a few genetic variants associated to FHA predisposition, suggest that epigenetic factors may represent additional candidates underlying FHA pathogenesis. Recent studies on mice have reported a pivotal role of epigenetics in controlling GnRH neuron ontogenesis, through the coordinated actions of *Dnmt3b*, *Tet1* and *Ezh2* on the *Fgf8* transcription (49). Moreover, *in vitro* studies further highlighted that the GnRH gene responds to external stimuli by modulating chromatin modifications also in mature GnRH neurons (50). This evidence suggests that epigenetics is a major molecular regulator of GnRH neuron development and function, and that the deregulation of these mechanisms may affect the activity of the HPG axis and the reproductive capacity.

Several studies analyzed the epigenetic profiling in amenorrheic women, mainly with AN, thus highlighting several differentially methylated genes. In particular, *LEP* and *LEPR* methylation levels were lower in AN women compared to controls (51) (**Figure 2**). Besides, lower DNA methylation of the *LEP* gene was associated with a significant hypermethylation during psychotherapeutic treatment and full recovery in AN patients (51), thus suggesting a predictive value of *LEP* methylation in identifying patients with a higher probability of recovery after treatment (51). Also hypomethylation of the *GHS-R1A* gene, that encodes for the ghrelin receptor, has been reported in AN patients, supporting the effect of environment on satiety and ghrelin resistance (52), and a possible effect of this epigenetic alterations in FHA patients (**Figure 2**).

GnRH secretion is controlled also by the activity of specific transcription factors. In particular, EAP1 (Enchanced At Puberty 1) is a transcription factor with a dual activity on the GnRH gene: it activates GnRH transcription and, at the same time, inhibits the expression of the preproenkephalin, that represses GnRH secretion. A polymorphic site in the 5-UTR of the gene has been associated with a higher risk for amenorrhea in non-human primate models (53), thus linking the epigenetic control of GnRH with common genetic variants that confer a higher risk for FHA (**Figure 2**).

Recently, growing findings have unveiled the central position of miRNAs as key regulators of GnRH secretion and consequent pituitary activation. Some miRNAs (miR-132, miR-212, miR-361-3p) have been reported to be induced by GnRH (54, 55) and play a role in the gonadotropin pathways, by directly targeting the 3’-UTR of LH and FSH transcripts or downregulating the expression of specific transcription factors (56) (**Figure 2**). The expression profiling of miRNAs in peripheral blood is emerging as a promising tool to monitor the effect of hormonal therapy in FHA. Studies on animal models have, indeed, demonstrated that kisspeptin-based hormonal therapy stimulates gonadotropin secretion and the altered expression of specific miRNAs in plasma (57). *In silico* prediction of targeted transcripts highlighted that kisspeptin-induced miRNAs may affect cell transport, structural and functional cell polarity, neural networks and intracellular trafficking, in addition to DNA methylation and sphingolipid metabolism. These studies, thus, open new research venues to identify the involvement of miRNAs in FHA and their possible use as peripheral biomarkers to monitor the effect of therapies. Nonetheless, microRNAs are not in clinical use yet, mainly due to technological limitations, including the lack of assay standardization and reproducibility (58).

## iPSCs to study the molecular mechanisms underlying amenorrhea

Most studies focused on the dissection of the neuroendocrine mechanisms leading to GnRH inhibition and FHA in presence of stress have been performed on animal models (5, 59, 60). However, this approach cannot fully recapitulate the complexity of the disease, given the physiological differences in estrous and menstrual cycles, the failure of reproduce in animal stressful conditions comparable to humans, and the higher complexity of response to stress in human that also encompasses self-awareness. In addition, since FHA is a multifactorial disease, animal models do not allow the comprehensive investigation of (epi)genetic and environmental factors associated with FHA. For these reasons, induced pluripotent stem cells (iPSCs) from FHA patients, that can be differentiated into GnRH neurons, are a promising tool to deepen the molecular bases of FHA, and *in vitro* investigate the effect of predicted predisposing variants and new therapeutic strategies.

In the last years, the generation of iPSC lines combined with the CRISPR–Cas9 technology has led to the generation and characterization of GnRH neurons, that are promising cell models to dissect signaling pathways and gene regulatory networks involved in human GnRH neuron development and function (61, 62). iPSCs from patients with AN have been already used to study changes in gene expression profiles that may occur in AN, highlighting that most of differentially expressed genes belong to the tachykinin receptor pathway and estrogen response (63), thus suggesting their possible application also to study the pathomechanisms underlying FHA. iPSCs may be also used to test therapies targeting not only GnRH release, but also the serotoninergic and dopaminergic neurotransmission, in order to improve the behavioral features of FHA, taking into account the personal response mediated by the epigenetic profile of each patient.

## Conclusions

Recent evidence highlighted that the establishment of FHA is variable among women, because it is a complex disease whose phenotypic appearance is influenced by several factors, including stress, behavioral habits and (epi)genetics, that together impact on the regulation of the HPG axis.

For this reason, (epi)genetic variants possibly associated to the personal predisposition to FHA should be investigated among genes that control the release and function of HPG-related hormones, and among genes involved in the interconnection between the environment and stress response.

The identification of rare genetic and epigenetic variants associated with FHA may have important clinical implications, as they may represent druggable targets for personalize medicine. In particular, germ-line variants may improve the clinical stratification of patients according to the patient- specific molecular profile, whereas epigenetic specific signatures, based on their dynamic nature, may represent valuable peripheral biomarkers for the diagnosis of the disease and for monitoring the effect of pharmacological and psychological therapies.

## Author contributions

FL critically revised the literature and wrote the manuscript; GE, MG, and GV provided support for the clinical content of the manuscript and critically revised the review; MM supervised and edited the manuscript. All authors contributed to the article and approved the submitted version.

## Acknowledgments

The authors acknowledge the University of Milan (through the APC initiative) for covering open access publication fees.

## Conflict of interest

The authors declare that the research was conducted in the absence of any commercial or financial relationships that could be construed as a potential conflict of interest.

## Publisher’s note

All claims expressed in this article are solely those of the authors and do not necessarily represent those of their affiliated organizations, or those of the publisher, the editors and the reviewers. Any product that may be evaluated in this article, or claim that may be made by its manufacturer, is not guaranteed or endorsed by the publisher.
